# Association of Rare *PTGIS* Variants With Susceptibility and Pulmonary Vascular Response in Patients With Idiopathic Pulmonary Arterial Hypertension

**DOI:** 10.1001/jamacardio.2020.0479

**Published:** 2020-04-01

**Authors:** Xiao-Jian Wang, Xi-Qi Xu, Kai Sun, Ke-Qiang Liu, Su-Qi Li, Xin Jiang, Qin-Hua Zhao, Lan Wang, Fu-Hua Peng, Jue Ye, Yan Wu, Rui Jiang, Jin Zhang, Wei Huang, Wen-Bin Wei, Yi Yan, Jing-Hui Li, Qian-Qian Liu, Sheng Li, Yong Wang, Shu-Yang Zhang, Xue Zhang, Zhi-Cheng Jing

**Affiliations:** 1 State Key Laboratory of Cardiovascular Disease, FuWai Hospital, National Center for Cardiovascular Diseases, Chinese Academy of Medical Sciences and Peking Union Medical College, Beijing, China; 2 Department of Cardiology, Peking Union Medical College Hospital, Chinese Academy of Medical Sciences and Peking Union Medical College Hospital, Beijing, China; 3 McKusick-Zhang Center for Genetic Medicine, State Key Laboratory of Medical Molecular Biology, Peking Union Medical College, Institute of Basic Medical Sciences, Chinese Academy of Medical Sciences, Beijing, China; 4 Department of Cardiopulmonary Circulation, Shanghai Pulmonary Hospital, Tongji University School of Medicine, Shanghai, China; 5 Ministry of Education (MOE) Key Laboratory of Bioinformatics, Bioinformatics Division, Beijing National Research Center for Information Science and Technology, Department of Automation, Tsinghua University, Beijing, China; 6 Cardiovascular Research Center, Xi’an Jiaotong University School of Basic Medical Sciences, Xi’an, China; 7 Department of Cardiology, The First Affiliated Hospital of Chongqing Medical University, Chongqing Medical University, Chongqing, China; 8 Cardiovascular Center, The Eighth Affiliated Hospital, Sun Yat-sen University, Shenzhen, China; 9 Shanghai Institute for Advanced Immunochemical Studies, ShanghaiTech University, Shanghai, China; 10 Department of Respiratory Medicine, Beijing Shijitan Hospital, Capital Medical University, Beijing, China

## Abstract

**Importance:**

Idiopathic pulmonary arterial hypertension (IPAH) is a fatal disease with high heritability; however, the bone morphogenetic protein receptor 2 (*BMPR2*) gene only accounts for 17% of IPAH. The genetic basis of IPAH needs further investigation.

**Objective:**

To identify novel IPAH susceptibility genes other than *BMPR2*.

**Design, Setting, and Participants:**

This 2-stage, case-control genetic association study enrolled 230 patients with IPAH from 2 referral pulmonary hypertension centers in China. Eligible patients had no *BMPR2* variants and were compared with 968 healthy control participants. Data were collected from January 1, 2000, to July 31, 2015, and analyzed from August 1, 2015, to May 30, 2018.

**Exposures:**

*PTGIS* rare variants.

**Main Outcomes and Measures:**

Whole-genome sequencing was performed to identify putative IPAH genes in a discovery cohort, with validation in an independent referral cohort. Correlation of genotype and hemodynamic characteristics was then evaluated at baseline and after pulmonary vasodilator testing. Functional assessments were conducted to analyze the effects of identified genetic variants on transcript splicing, enzymatic activity, and endothelial cell phenotypes.

**Results:**

Among 230 patients with IPAH (164 female [71.3%]; mean [SD] age, 34 [18] years), an enrichment of rare variants in a gene encoding prostacyclin synthase (*PTGIS*) was identified in the discovery cohort. The association of *PTGIS* rare variants with IPAH was confirmed in the replication cohort. In the combined data set, *PTGIS* rare variants were found in 14 of 230 cases (6.1%) and 8 of 968 controls (0.8%) (odds ratio, 7.8; 95% CI, 3.2-18.8; *P* = 5 × 10^−6^, logistic regression). Compared with patients without *PTGIS* variants, inhaled iloprost induced a more significant decrease of pulmonary vascular resistance (difference in the least square mean, −21.7%; 95% CI, −31.4% to −12.0%; *P* < .001, linear regression model) and an increase of cardiac index (difference in the least square mean, 18.3%; 95% CI, 8.8%-27.8%; *P* < .001, linear regression model) in patients with *PTGIS* variants. The minigene assay indicated that the c.521 + 1G>A variant resulted in aberrant messenger RNA transcripts. The functional studies showed that the 2 missense rare variants (R252Q and A447T) resulted in a decrease in prostacyclin production and increased cell death of pulmonary microvascular endothelial cells.

**Conclusions and Relevance:**

This study identified 3 rare loss-of-function variants in the *PTGIS* gene from 2 independent cohorts with IPAH. The genetic variants of *PTGIS* predispose pulmonary vascular responses to the iloprost stimulation. These findings suggest that *PTGIS* variants may be involved in the pathogenesis of IPAH.

## Key Points

**Question:**

What is the novel susceptibility gene for idiopathic pulmonary arterial hypertension?

**Findings:**

In this 2-stage genetic association study of 230 patients with idiopathic pulmonary arterial hypertension, heterozygous rare *PTGIS* variants were first found significantly overrepresented in 6.1%, conferring 7.8 higher odds of pulmonary arterial hypertension. In addition, patients carrying rare *PTGIS* variants were more responsive to iloprost stimulation than those without such variants.

**Meaning:**

The rare variants of the *PTGIS* gene appear to contribute higher susceptibility to idiopathic pulmonary arterial hypertension, and screening of *PTGIS* variants may help improve personalized treatment of these patients.

## Teaser

This genetic association study assesses novel susceptibility genes other than *BMPR2* in patients with idiopathic pulmonary arterial hypertension.

## Introduction

Pulmonary arterial hypertension (PAH) is a rare disease with an estimated prevalence of 15 to 50 cases per 1 million adults.^1^ Approximately 40% of PAH occurs in individuals without any family history or predisposing conditions, when it is termed *idiopathic PAH* (IPAH).^2,3^ The pathogenesis of IPAH is complicated and largely unknown. The prognosis is still poor, with a 5-year survival rate at 50%.^4,5^

Genetic variants are closely associated with PAH.^6^ To date, at least 17 PAH risk genes have been reported.^7^ However, all these known risk genes explain only a small proportion of cases of IPAH. Taking the most predominant causal gene, bone morphogenetic protein receptor type 2 (*BMPR2* [OMIM 600799]), as an example, its variants only account for 17% of patients with IPAH.^8^ For other known predisposing genes, such as *CAV1* and *KCNK3*, the genetic variants are rare in patients with IPAH.^9,10^

In addition to the unexplained etiology of IPAH, the reported PAH disease genes are not relevant to any pathways corresponding to important therapeutic targets. For example, the dysregulation of the prostacyclin metabolic pathway in patients with PAH has long been documented. The production of prostacyclin is significantly decreased during the onset of PAH.^11,12^ Four drugs (epoprostenol sodium, iloprost, treprostinil sodium, and beraprost sodium) targeting the prostacyclin pathway have been developed and recommended for the treatment of advanced PAH.^13^ However, whether the genetic variants in the prostacyclin pathway influence the initiation and development of IPAH is largely unknown. Thus, large-scale genetic analyses using cutting-edge technology are crucial for identification of additional IPAH-susceptible loci, especially those involved in clinical management of IPAH.

Liu et al^14^ have previously reported a mutation rate of 14.5% for *BMPR2* in Chinese patients with IPAH. In the present study, we sought to perform whole-genome sequencing (WGS) in a cohort of patients with IPAH and without the *BMPR2* variants to identify novel genetic variants associated with IPAH, with subsequent evaluation of pulmonary vasodilator responsiveness in patients with the identified variants, followed by functional characterization of the genetic variants.

## Methods

### Study Population

The study participants were patients with incident IPAH who were recruited from 2 pulmonary hypertension referral centers in China: Shanghai Pulmonary Hospital, Tongji University School of Medicine, Shanghai, and FuWai Hospital, Chinese Academy of Medical Sciences, Beijing. Baseline hemodynamic measurements and pulmonary vasodilator testing were performed in patients with IPAH before starting pulmonary vasodilator therapy. Pulmonary arterial hypertension was diagnosed as a mean pulmonary arterial pressure of at least 25 mm Hg at rest, a pulmonary artery wedge pressure of no greater than 15 mm Hg, and pulmonary vascular resistance of greater than 3 Wood units.^15^ The diagnosis of IPAH was made by at least 2 experienced PAH experts (X.-Q.X., X.J., L.W., X.-C.J.). Patients with known causes of PAH listed in the classification of the current guideline were excluded, including hereditary hemorrhagic telangiectasia, drug-induced PAH, connective tissue disease, congenital heart disease, and chronic thromboembolic pulmonary hypertension.^15^ The participants were then screened for *BMPR2* point mutations and large rearrangements by direct Sanger sequencing and multiplex ligation-dependent probe amplification technology.^16^ Individuals carrying any *BMPR2* variant were excluded from the study. Patients with a familial history of PAH were also excluded. The study protocol was approved by the ethics committees of Shanghai Pulmonary Hospital and FuWai Hospital. All participants provided written informed consent. This study followed the Strengthening the Reporting of Genetic Association Studies (STREGA) reporting guideline.

The study consisted of a discovery stage and a replication stage (eFigure 1 in the Supplement). In the discovery stage, the WGS was performed for 42 patients with IPAH without any *BMPR2* variants. The replication stage involved 188 unrelated patients with IPAH and 968 healthy control participants. In the first control cohort, 460 participants were recruited from the health examination center in Peking University Hospital, and genetic variants in candidate genes were screened by Sanger sequencing. In the second control cohort, 508 participants were enrolled from the Novo-Zhonghua Genomes Database (an in-house, Chinese population–specific reference database) maintained by Novogene, and genetic variants were assessed using whole-exome sequencing.

All participants were officially registered as Han Chinese, and ethnicity was confirmed by identity-by-descent analysis in 42 cases who underwent WGS and 508 controls from Novo-Zhonghua Genomes Database. The kinship between the 42 cases and 508 controls was analyzed using King software.^17^ Methods for WGS, Sanger sequencing, right heart catheterization, pulmonary vasodilator testing, plasmid construction, cell culture and transfection, measurement of 6-Keto–prostaglandin F_1α_ levels, and the functional assessments are detailed in the eMethods in the Supplement.

### Statistical Analysis

Data were collected from January 1, 2000, to July 31, 2015, and analyzed from August 1, 2015, to May 30, 2018. Results were recorded as percentages, median (interquartile range), or mean (standard error of mean or SD), as indicated. The normality of data distribution was assessed using Kolmogorov-Smirnov test. A χ^2^ test or Fisher exact test was applied to compare qualitative variables and genotype/allele frequencies. For quantitative variables of clinical characteristics between the discovery and replication cohorts, statistical significance was determined using an unpaired *t* test or 1-way analysis of variance. The association between the genetic variants and disease status was assessed using logistic regression. The effect of genetic variants on clinical phenotype was analyzed by the linear regression model, which had genotype and baseline measurement as predictors. All the statistical testing was 2 sided. Results were considered statistically significant at a level of *P* < .05. All analyses were performed with PASW Statistics, version 18.0 (SPSS, Inc).

## Results

### Study Patients

In the discovery cohort, 42 patients with IPAH (including 10 pediatric patients) were recruited from Shanghai Pulmonary Hospital and FuWai Hospital. For the replication cohort, 188 patients (including 27 pediatric patients) were enrolled. In total, 230 patients with IPAH were recruited from the 2 national referral centers (Table 1) (164 female [71.3%] and 66 male [28.7%]; mean [SD] age, 34 [18] years).

**Table 1.** Demographic, Clinical, and Invasive Hemodynamic Characteristics of the 2 IPAH Cohorts

Demographic characteristics	IPAH cohort^a^	
	WGS discovery (n = 42)	Replication (n = 188)
Age at diagnosis, y	23 (10)	37 (18)^b^
Female, No. (%)	34 (81.0)	130 (69.1)
6-min walking distance, m	425 (96)	379 (139)^b^
WHO functional class, No. (%)^c^		
I and II	16 (38.1)	66 (35.1)
III and IV	26 (61.9)	122 (64.9)
mPAP, mm Hg	55 (12)	62 (18)
PAWP, mm Hg	7.7 (3.8)	8.6 (4.3)
PVR, Wood units	14.2 (7.1)	15.6 (8.8)
RAP, mm Hg	7.2 (5.8)	7.7 (6.0)
Cardiac index, L/min/m^2^	2.7 (1.3)	2.6 (1.0)
SvO_2_, %	64 (19)	63 (11)

Abbreviations: IPAH, idiopathic pulmonary arterial hypertension; mPAP, mean pulmonary artery pressure; PAWP, pulmonary artery wedge pressure; PVR, pulmonary vascular resistance; RAP, right atrial pressure; SvO_2_, mixed venous oxygen saturation; WGS, whole-genome sequencing; WHO, World Health Organization.

^a^ Unless otherwise indicated, data are expressed as mean (SD).

^b^ Compared with the WGS discovery cohort, *P* < .05, unpaired *t* test.

^c^ Range from I to IV, with higher numbers indicating greater functional limitations.

### Whole-Genome Sequencing

Whole-genome sequencing was performed in the discovery cohort. To identify new pathogenic variants under the dominant model, we analyzed the genome data and screened out potential deleterious heterozygous variants with the minor allele frequencies of less than 0.5% in 4 variant databases and absent in the Chinese population from the 1000 Genomes Project (eTable 1 in the Supplement). A total of 1986 rare variants were predicted to affect 1772 candidate genes. Most of these genes (1551 [87.5%]) harbored 1 variant in a single case. Only 15 genes (0.8%) harbored rare variants shared by 3 or more individuals (eTable 2 in the Supplement). Four genes, including *PTGIS* (Gene ID 5740), *MACF1* (Gene ID 23449), *GTF3C1* (Gene ID 2975), and *ABCA3* (Gene ID 21), are abundantly expressed in the lung (eTable 2 in the Supplement). Given the key role of the gene encoding prostacyclin synthase (*PTGIS*) in prostacyclin production, we hypothesized that the clinical outcomes from *PTGIS* variants may be more relevant than those from the other 14 genes listed in eTable 2 in the Supplement. Therefore, *PTGIS* was chosen for further validation.

### Validation of the Genetic Susceptibility of *PTGIS* With IPAH

In the WGS cohort, we identified 3 patients carrying 2 heterozygous single-nucleotide variants of *PTGIS* (Genebank NM_000961.3), including 1 with c.521 + 1G>A and 2 with c.1339G>A. The c.521 + 1G>A variant was located at the +1 position of the splice donor site of intron 4. The missense variant, c.1339G>A, causes the amino acid substitution p.Ala447Thr (A447T; rs146531327) (eFigures 2A and 3 in the Supplement). We have rechecked the exome data of the 3 patients for 17 genes known to be associated with PAH (*BMPR2*, *EIF2AK4*, *TBX4*, *ATP13A3*, *GDF2*, *SOX17*, *AQP1*, *ACVRL1*, *SMAD9*, *ENG*, *KCNK3*, *CAV1*, *SMAD4*, *SMAD1*, *KLF2*, *BMPR1B*, and *KCNA5*). None of the patients had any deleterious rare variants in the known PAH-related genes.

In the replication cohort, we screened all exons and splicing sites of *PTGIS* by Sanger sequencing (eTable 3 in the Supplement) and detected another 6 cases carrying the variant A447T. The prevalence for *PTGIS* A447T variant was comparable between the discovery (2 of 42 [4.8%]) and replication (6 of 188 [3.2%]) cohorts (χ^2^_1_ = 0.0013; *P* = .97, continuity-adjusted χ^2^ test). In addition, we detected another rare missense *PTGIS* variant, c.755G>A (p.Arg252Glu [R252Q]; rs759344518) in 5 cases in the replication cohort (eFigures 2A and 3 in the Supplement). Given the variant R252Q passed the population frequency filters (minor allele frequency, <0.5%, and absent in Chinese population from the 1000 Genomes Project), it was included in further analysis. The copy number variations and structure variations of *PTGIS* were analyzed by in silico tools in the discovery cohort and by real-time polymerase chain reaction analysis in the replication cohort. No such variant was found (eFigure 4 in the Supplement). In total, we identified 14 patients (including 2 pediatric cases) carrying *PTGIS* rare variants. No significant difference was observed in the genetic variant rate between pediatric and adult cases with PAH (2 of 37 [5.4%] and 12 of 193 [6.2%], respectively; *P* > .99, continuity-adjusted χ^2^ test).

To confirm the association of *PTGIS* rare variants with IPAH, all exons and flanking intronic sequences of *PTGIS* were sequenced in the control cohort. The genetic structure was homogeneous between cases with IPAH and controls (eFigure 5 in the Supplement). The kinship analysis was performed using the exome data from 42 cases and 508 controls. More than 99.9% of the kinship values between cases with IPAH and controls were less than 0.0884, and the maximum kinship value was 0.1400, suggesting that participants in the study were unrelated. For the 188 cases and 460 controls who did not have the exome data, we carefully checked the recoded ancestry information and confirmed that they were unrelated individuals. In total, we identified 6 participants carrying the variant A447T and 2 with the variant R252Q, and no splicing variant was detected in the control cohort. The R252Q A allele (5 of 230 [2.2%] vs 2 of 968 [0.2%]; *P* = .005, logistic regression) and A447T A allele (8 of 230 [3.5%] vs 6 of 968 [0.6%]; *P* = .001, logistic regression) were associated with a higher risk of IPAH (Table 2). In terms of the genetic burden, the rare variant frequency was 6.1% (14 of 230) in the patients with IPAH compared with 0.8% (8 of 968) in the control group (odds ratio, 7.8; 95% CI, 3.2-18.8; *P* = 5.0 × 10^−6^, logistic regression) (Table 2). These results strongly suggest that the *PTGIS* locus may contribute to the pathogenesis and pathophysiology of IPAH.

**Table 2.** Association of *PTGIS* Rare Variants With Pulmonary Arterial Hypertension

Nucleotide change	Cohort, No. of participants		No. (%) of participants		*P* value^a^	OR (95% CI)^a^
	Discovery (n = 42)	Replication (n = 188)	Combined cases (n = 230)	Controls (n = 968)		
c. 521 + 1 G>A	1	0	1 (0.4)	0	NE	NE
c. 755 G>A	0	5	5 (2.2)	2 (0.2)	.005	10.7 (2.1-55.7)
c. 1339 G>A	2	6	8 (3.5)	6 (0.6)	.001	5.8 (2.0-16.8)
Combined	3	11	14 (6.1)	8 (0.8)	5 × 10^−6^	7.8 (3.2-18.8)

Abbreviations: NE, not estimable; OR, odds ratio; *PTGIS*, prostacyclin synthase.

^a^ Calculated with the logistic regression model.

All the 3 *PTGIS* variants were located in the conserved region (eFigure 2B in the Supplement) and were predicted to be deleterious by in silico analysis (eTable 4 in the Supplement). According to the crystal structure of *PTGIS*, A447 is located at the highly conserved N-terminus of the alpha L helix of the protein, which is within 3.5 Å of the active site (eFigure 2C in the Supplement). The A447T produces a larger side chain, which could hinder the ligand-binding space. Moreover, a swap of nonpolar alanine for polar threonine will alter the electrostatics of the binding site, which is likely to cause a change in the *PTGIS* catalytic activity (eFigure 2D in the Supplement).

### Clinical Phenotypes of Cases With *PTGIS* Rare Variants

The clinical, functional, and hemodynamic characteristics of the 14 patients with IPAH carrying the *PTGIS* variants are shown in eTable 5 in the Supplement. Noticeably, these patients were diagnosed with IPAH at a young age (median, 26 [range, 17-70] years). Significant sex bias was also observed in these 14 patients, with a female-to-male ratio of 6:1.

**Figure 1.**
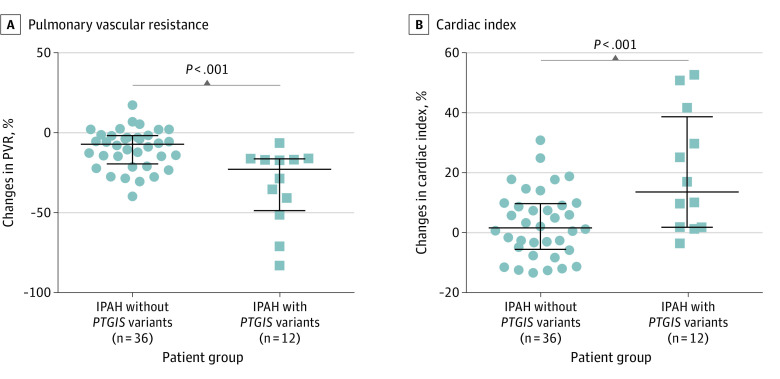
Comparison of Acute Hemodynamic Responses to Aerosolized Iloprost in Patients With Idiopathic Pulmonary Arterial Hypertension (IPAH) With or Without Prostacyclin Synthase (*PTGIS*) Gene Variants Iloprost inhalation induced more significant decrease of pulmonary vascular resistance (PVR) and increase of cardiac index in patients with variants in *PTGIS*. The data were analyzed by the linear regression model.

To determine whether the identified *PTGIS* variants were correlated with different responses to prostacyclin, we compared the acute hemodynamic response to iloprost, a synthetic analogue of prostacyclin, between 12 patients with *PTGIS* variants (mean [SD] age, 32 [19] years; female-to-male ratio, 10:2) and 36 age- and sex-matched patients without *PTGIS* variants (mean [SD] age, 32 [8] years; female-to-male ratio, 31:5). Two patients with *PTGIS* variants were excluded from this analysis because they did not undergo iloprost testing at baseline. The changes from baseline were analyzed by a linear regression model, which had genotype and baseline measurement as predictors. As shown in Figure 1 and eTable 6 in the Supplement, the pulmonary vascular resistance decreased (difference in the least square mean, −21.7%; 95% CI, −31.4% to −12.0%; *P* < .001, linear regression model), and the cardiac index increased (difference in the least square mean, 18.3%; 95% CI, 8.8% to 27.8%; *P* < .001, linear regression model) more significantly in patients with *PTGIS* variants than those without. Thus, genetic variants of *PTGIS* predisposed pulmonary vascular responses to the iloprost stimulation.

### Interference of *PTGIS* Rare Variants With Enzyme Function

It has been shown previously that *PTGIS* is downregulated in the lung vasculature of patients with severe PAH.^18^ To confirm this finding, we examined the expression level of *Ptgis* in 3 experimental PAH rat models. Compared with healthy control rats, *Ptgis* messenger RNA levels significantly decreased in the monocrotaline-treated lungs (50% reduction; *P* = .03, analysis of variance), lungs with hypoxia (41% reduction; *P* = .04, analysis of variance), and lungs with sugen treatment (SU-5416) plus hypoxia (55% reduction; *P* = .02, unpaired *t* test) (eFigures 6 and 7 in the Supplement). These results were consistent with the decrease of PTGIS in the lungs of patients with PAH, indicating that an optimal level of PTGIS is necessary for functional homeostasis of the pulmonary vasculature.

**Figure 2.**
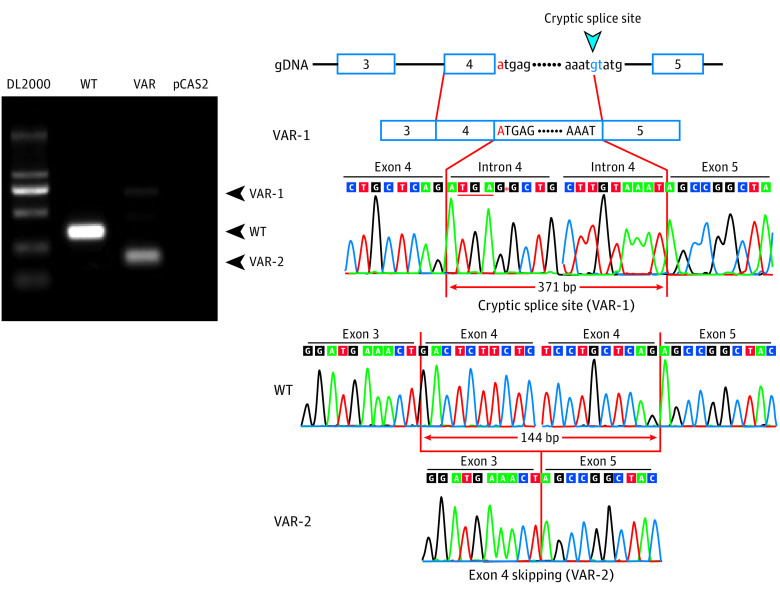
Functional Studies of the 3 Variants of the Prostacyclin Synthase (*PTGIS*) Gene Minigene assay for the c.521 + 1G>A variant. The left panel shows the reverse-transcription polymerase chain reaction analysis of HEK293T cells transfected with pCAS2 reporter minigenes for the wild-type (WT) or the c.521 + 1G>A variant. Two distinct bands were seen on the lane of the variant reporter minigene. Sanger sequencing showed that the bright short band from pCAS2-*PTGIS*-Ex3-5-VAR was an aberrant transcript with complete exon 4 skipping, and the dim long band resulted from the activation of a cryptic splice site in intron 4 (right panel).

The rare variant c.521 + 1G>A replaced the almost invariant GU (guanine-uracil) with an AU (adenine-uracil). To determine the effect of c.521 + 1G>A on the *PTGIS* transcription, we performed a minigene assay (Figure 2 and eFigure 8 in the Supplement). When transfected into the HEK293T cells, the mutant plasmid produced 2 distinct transcripts, including 1 with exon 4 skipping (dim band in Figure 2, right panel) and 1 resulting from an activation of a cryptic splice site in the intron 4 (bright band in Figure 2, middle panel). Exon 4 skipping caused an in-frame deletion of 144 base pairs (bp) in the *PTGIS* transcript (Figure 2, right panel) that was predicted to have a 48-amino acid deletion (p.Thr127_Arg174del) within the region homologous to cytochrome P450 superfamily according to the CATH database, which could lead to a compromised enzymatic activity. Furthermore, 1 cryptic donor 371 bp downstream of exon 4 was activated, resulting in a premature termination codon immediately following the transcript product from exon 4 (Figure 2, middle panel). Therefore, the rare variant c.521 + 1G>A resulted in 2 types of aberrant messenger RNA transcript, and both transcripts may be translated into loss-of-function PTGIS protein.

**Figure 3.**
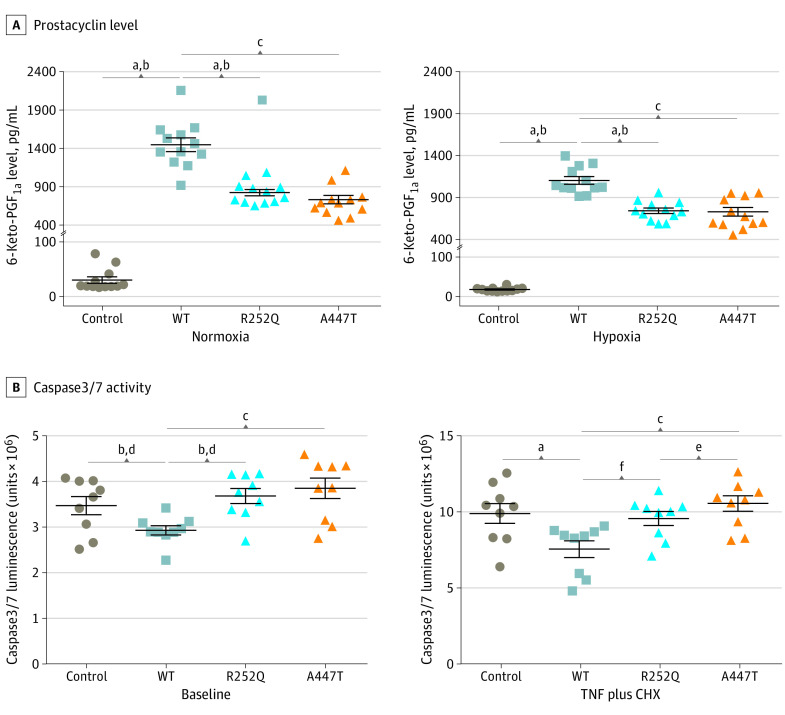
Functional Studies for the R252Q and A447T Variants of the Prostacyclin Synthase (*PTGIS*) Gene All experiments were repeated in 3 to 4 times. A, After plasmid transfection, the 6-keto–prostaglandin F_1α_ (PGF_1α_) concentration was measured in pulmonary microvascular endothelial cells (PMECs) culture supernatant. B, The effect of the variants of *PTGIS* on apoptosis in PMECs was assessed by caspase 3/7 activity after stimulation with tumor necrosis factor (TNF) and cycloheximide (CHX). *P* values were calculated using analysis of variance. ^a^*P* < .001, control vs WT. ^b^*P* < .001, control vs R252Q. ^c^*P* < .001, WT vs A447T. ^d^*P* = .007, control vs WT. ^e^*P* < .001, WT vs R252Q. ^f^*P* < .001, R252Q vs A447T.

In the lung, PTGIS is predominantly localized in pulmonary endothelial cells,^18^ where it plays a critical role in regulating endothelial cell viability, apoptosis, and integrity.^19,20^ To investigate the consequences of the 2 missense *PTGIS* rare variants, we transfected the pulmonary microvascular endothelial cells (PMECs) with *PTGIS* wild-type (WT) and variant plasmids and analyzed the effect of the *PTGIS* variants on the enzyme activity and function by measuring 6-keto–prostaglandin F_1α_ production. Immunoblot analysis showed similar protein levels in the total lysates of PMECs expressing the WT or 2 missense variant subunits, indicating that the variant proteins had similar stability to the WT protein (eFigure 9 in the Supplement). Both R252Q and A447T variants had significantly reduced PTGIS activities. At the baseline level, both variants generated only 57% (*P* < .001) and 50% (*P* < .001) of 6-keto–prostaglandin F_1α_ levels when compared with WT transfected cells. Under the hypoxic condition, the levels decreased by 33% (*P* < .001) and 34% (*P* < .001), respectively (Figure 3).

We compared PMEC viability caused by ectopic overexpression of WT PTGIS or mutants. Wild-type PTGIS was first overexpressed in PMECs and then cultured under hypoxic or normoxic conditions. As shown in eFigure 10 in the Supplement, cell viability increased by 20% and 30% when compared with empty vector-transfected cells under respective conditions. Although the exogenously expressed R252Q variant affected little of the cell viability, the A447T variant reduced cell viability at the baseline condition (9.6%; *P* < .001) and under hypoxic conditions (12.0%; *P* = .01) when compared with WT vector-transfected cells (eFigure 10 in the Supplement). Moreover, R252Q and A447T variants attenuated the antiapoptosis effect of WT PTGIS either at baseline or in response to the stimulation of tumor necrosis factor and cycloheximide (Figure 3). We next assessed the angiogenic potential. In vitro tube formation by PMECs was enhanced by the overexpression of WT *PTGIS*. However, the PTGIS-enhanced tube formation was not seen in PMECs expressing either A447T or R252Q variants (eFigure 11 in the Supplement). Together these studies indicated that the 2 missense rare variants of *PTGIS* impaired the endothelial cell–protective function of PTGIS.

## Discussion

Using data from WGS of patients with IPAH, we demonstrated that *PTGIS* might be a PAH susceptibility gene. Importantly, these patients lacked any *BMPR2* variants, which suggests that the IPAH susceptibility of *PTGIS* variants is independent of *BMPR2.* The rare *PTGIS* variants identified in the discovery and replication cohorts conferred a greater odds ratio of 7.8-fold to develop IPAH. Furthermore, patients with *PTGIS* variants were more sensitive to iloprost stimulation. Functional studies revealed that the *PTGIS* splicing variant interfered with its transcription, and the 2 missense variants caused impaired enzyme activity, resulting in decreased viability of PMECs. These findings suggest that the rare loss-of-function variants of *PTGIS* may contribute to the genetic etiology of IPAH (eFigure 12 in the Supplement).

The advances in sequencing technology accelerate our understanding for human diseases. Recently, several novel PAH disease gene or susceptibility loci have been identified using WGS.^21,22^ However, identification of variants that are specifically associated with PAH is still difficult. To address this caveat, we first excluded the detrimental effect of *BMPR2* variants. To minimize variability, we used the strategy of rare variant enrichment with stringent filtering criteria in the discovery cohort and found *PTGIS* as a candidate gene. We subsequently genotyped an additional 188 patients with IPAH and determined the association of the 3 risk alleles of *PTGIS* with IPAH.

Under physiological conditions, constitutive PTGIS couples with thromboxane-A synthase to regulate the prostacyclin–thromboxane A_2_ metabolism. The balance of prostacyclin–thromboxane A_2_ state is necessary for physiological homeostasis and functional endothelium.^23^ In contrast, under pathological conditions (eg, hypoxia, increased shear stress, and injury), the decreased expression of PTGIS in pulmonary endothelial cells^18^ leads to a deficiency in prostacyclin and a supraphysiological level of thromboxane A_2_.^11,12^ Such a dysregulated prostacyclin axis would cause the apoptosis of lung endothelial cells and the remodeling of the pulmonary vasculature.^24^ In the present study, we identified 3 rare *PTGIS* variants that are overrepresented in patients with IPAH and without *BMPR2* variants compared with healthy controls. Functional studies showed that all 3 rare variants exhibited an impaired function of PTGIS. Compared with R252Q, A447T is more proximal to the active site, which may explain why A477T was more deleterious than R252Q. Given the importance of PTGIS in the regulation of pulmonary vasculature tone, we reasoned that rare loss-of-function variants of *PTGIS* predispose to the risk of IPAH. Of note, the population minor allele frequency for A477T (0.31%) and R252 (0.16%) is higher in East Asian than in other populations (minor allele frequency, 0%-0.013% for R252Q, 0%-0.011% for A447T [gnomAD]). These findings may or may not only apply to individuals of East Asian ancestry, which needs further evaluation in future studies.

The inheritance of IPAH is autosomal dominant, whereas a female predominance is common. Compared to males, females with *BMPR2* variants are about 2.5-fold more susceptible to familial PAH. Among *ACVRL1* variant carriers who develop IPAH, the female-to-male ratio has been reported to be 3.5:1.0.^25^ In the present study, we also observed a sex bias in *PTGIS* rare variant carriers, with a female-to-male ratio of 6:1. The predominance of adult female carriers may be causative between sex hormones and *PTGIS*. Indeed, combined administration of estradiol-17β and progesterone to ovariectomized sheep increases levels of cytosolic phospholipase A_2_ and cyclooxygenase-1 in uterine artery endothelial cells. Consistently, combined hormone administration also increases PTGIS levels in uterine artery vascular smooth muscle cells.^26^ Further studies are warranted to confirm the clinical characteristics of sex bias with respect to *PTGIS* rare variant carriers.

The previous study by Stearman et al^27^ has shown that functional *PTGIS* promoter polymorphisms exert a protective effect on the penetrance of *BMPR2* variants. In the present study, our genetic data showed the significant deleterious effect of rare *PTGIS* variants on the development of IPAH independent of *BMPR2*. Given the strong association between rare *PTGIS* variants and IPAH, further studies are needed to assess whether these variants represent a true PAH disease state that is distinct from a grayer area or a potential contamination with secondary pulmonary hypertension. Moreover, our clinical data from acute pulmonary vasoreactivity testing demonstrate that rare *PTGIS* genetic variants are predictive of iloprost responsiveness, thereby providing a rationale for targeting the prostacyclin pathway as part of tailored therapies for managing patients with *PTGIS* variants.

### Limitations

Our study has several limitations. First, because all the involved patients in the 2 cohorts had sporadic PAH and the DNA samples from the relatives were not available, familial segregation of rare *PTGIS* variants with PAH could not be demonstrated. Second, rare variant testing (eg, burden tests) was not performed owing to the small sample size, and future studies to validate these findings will be needed to explore results using contemporary rare variant statistical testing. Third, the pulmonary vascular response to iloprost was only measured at the acute phase for a relatively low number of patients with IPAH, with or without rare *PTGIS* variants. Future study among larger cohorts will be crucial to test the variability and prognostic value of rare *PTGIS* variants for long-term prostacyclin treatment or the relevance to different therapeutic strategy.

## Conclusions

We have identified rare variants in the *PTGIS* gene that may represent a novel susceptibility factor for IPAH in Chinese patients. These variants may also modulate pulmonary vasodilator responsiveness to inhaled prostacyclin.

## Notes

Supplement. eFigure 1. Study Design eFigure 2. Analysis of the 3 Prostacyclin Synthase (PTGIS) Gene Variants eFigure 3. Verification of the 3 Rare Variants of *PTGIS* in IPAH Cases by Sanger Sequencing eFigure 4. Analysis of Structure Variants of *PTGIS* in the Replication Cohort by Real-Time PCR eFigure 5. Assessment of Genetic Structure of Cases and Controls eFigure 6. Downregulation of *Ptgis* mRNA in the Lungs of MCT and Hypoxia Rat Models eFigure 7. Downregulation of *Ptgis* mRNA in the Lungs of Sugen Plus Hypoxia Rat Models eFigure 8. Blueprint of the Minigene Plasmid Construction eFigure 9. Protein Blot Analysis eFigure 10. Cell Viability Assay eFigure 11. Representative Images of Tube Formation From Pulmonary Microvascular Endothelial Cells (PMECs) Transfected With the Plasmids for Wild-type (WT) and 2 Variants eFigure 12. Conceptual Representation of the Role of *PTGIS* Rare Variants in PAH eMethods. Sequencing and Analysis eTable 1. Filtering Strategy to Identify Rare Genetic Variants in Whole-Genome Sequencing Data eTable 2. Candidate Genes Identified Via WGS in the Discovery Cohort eTable 3. The PCR Primers for Amplifying the 10 Exons of the *PTGIS* Gene eTable 4. Rare Variants in the *PTGIS* Gene eTable 5. Clinical Characteristics of the 14 Patients With *PTGIS* Rare Variants eTable 6. The Influence of *PTGIS* Rare Variants on Pulmonary Vascular Response to Iloprost eReferences.
