# Supplementary material for: Association of Rare PTGIS Variants With Susceptibility and Pulmonary Vascular Response in Patients With Idiopathic Pulmonary Arterial Hypertension
Source: JAMA Cardiol. 2020 Apr 1;5(6):1–8. doi: 10.1001/jamacardio.2020.0479 (PMC7113838; doi:10.1001/jamacardio.2020.0479)
Supplement: Supplement. — eFigure 1. Study Design eFigure 2. Analysis of the 3 Prostacyclin Synthase (PTGIS) Gene Variants eFigure 3. Verification of the 3 Rare Variants of PTGIS in IPAH Cases by Sanger Sequencing eFigure 4. Analysis of Structure Variants of PTGIS in the Replication Cohort by Real-Time PCR eFigure 5. Assessment of Genetic Structure of Cases and Controls eFigure 6. Downregulation of Ptgis mRNA in the Lungs of MCT and Hypoxia Rat Models eFigure 7. Downregulation of Ptgis mRNA in the Lungs of Sugen Plus Hypoxia Rat Models eFigure 8. Blueprint of the Minigene Plasmid Construction eFigure 9. Protein Blot Analysis eFigure 10. Cell Viability Assay eFigure 11. Representative Images of Tube Formation From Pulmonary Microvascular Endothelial Cells (PMECs) Transfected With the Plasmids for Wild-type (WT) and 2 Variants eFigure 12. Conceptual Representation of the Role of PTGIS Rare Variants in PAH eMethods. Sequencing and Analysis eTable 1. Filtering Strategy to Identify Rare Genetic Variants in Whole-Genome Sequencing Data eTable 2. Candidate Genes Identified Via WGS in the Discovery Cohort eTable 3. The PCR Primers for Amplifying the 10 Exons of the PTGIS Gene eTable 4. Rare Variants in the PTGIS Gene eTable 5. Clinical Characteristics of the 14 Patients With PTGIS Rare Variants eTable 6. The Influence of PTGIS Rare Variants on Pulmonary Vascular Response to Iloprost eReferences. [file jamacardiol-5-677-s001.pdf]

Supplementary Online Content

Wang X-J, Xu X-Q, Sun K, et al. Association of rare *PTGIS* variants with susceptibility and pulmonary vascular response in patients with idiopathic pulmonary arterial hypertension. *JAMA Cardiol.* Published online April 1, 2020. doi:10.1001/jamacardio.2020.0479

eFigure 1. Study Design
eFigure 2. Analysis of the 3 Prostacyclin Synthase (*PTGIS*) Gene Variants
eFigure 3. Verification of the 3 Rare Variants of *PTGIS* in IPAH Cases by Sanger Sequencing
eFigure 4. Analysis of Structure Variants of *PTGIS* in the Replication Cohort by Real-Time PCR
eFigure 5. Assessment of Genetic Structure of Cases and Controls
eFigure 6. Downregulation of *Ptgis* mRNA in the Lungs of MCT and Hypoxia Rat Models
eFigure 7. Downregulation of *Ptgis* mRNA in the Lungs of Sugen Plus Hypoxia Rat Models
eFigure 8. Blueprint of the Minigene Plasmid Construction
eFigure 9. Protein Blot Analysis
eFigure 10. Cell Viability Assay
eFigure 11. Representative Images of Tube Formation From Pulmonary Microvascular Endothelial Cells (PMECs) Transfected With the Plasmids for Wild-type (WT) and 2 Variants
eFigure 12. Conceptual Representation of the Role of *PTGIS* Rare Variants in PAH
eMethods. Sequencing and Analysis
eTable 1. Filtering Strategy to Identify Rare Genetic Variants in Whole-Genome Sequencing Data
eTable 2. Candidate Genes Identified Via WGS in the Discovery Cohort
eTable 3. The PCR Primers for Amplifying the 10 Exons of the *PTGIS* Gene
eTable 4. Rare Variants in the *PTGIS* Gene
eTable 5. Clinical Characteristics of the 14 Patients With *PTGIS* Rare Variants
eTable 6. The Influence of *PTGIS* Rare Variants on Pulmonary Vascular Response to Iloprost
eReferences

This supplementary material has been provided by the authors to give readers additional information about their work.

© 2020 Wang X-J et al. *JAMA Cardiology*.

eFigure 1. Study Design

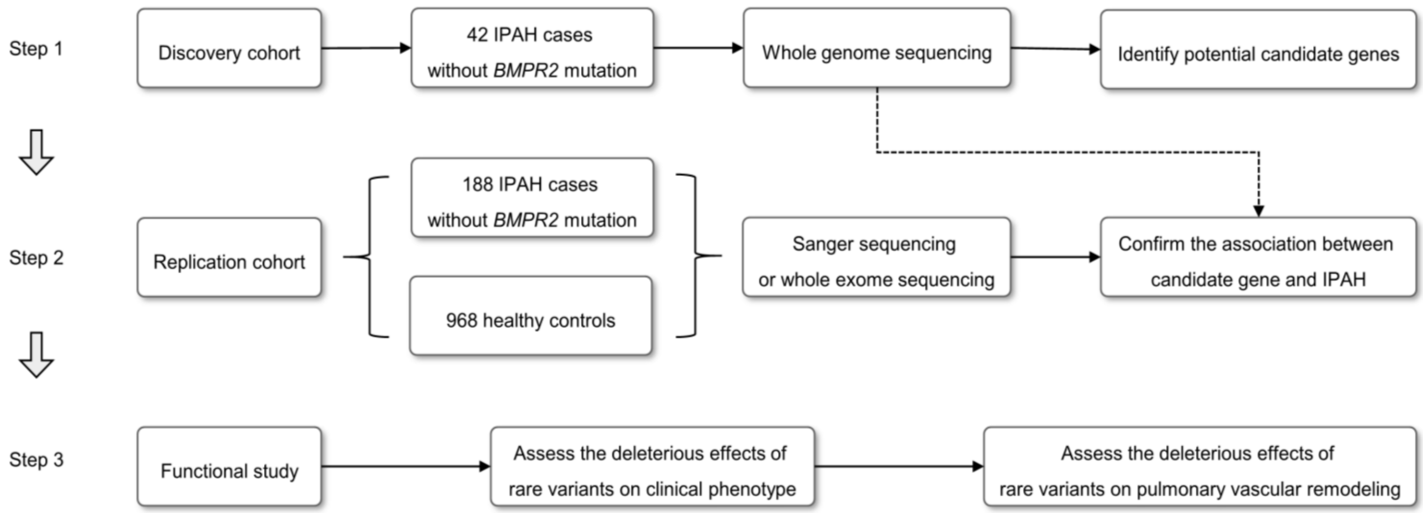

Flow diagrams illustrating the research strategy to identify novel PAH genes. *BMPR2* denotes bone morphogenetic protein receptor 2; IPAH denotes idiopathic pulmonary arterial hypertension.

© 2020 Wang X-J et al. *JAMA Cardiology*.

eFigure 2. Analysis of the 3 Prostacyclin Synthase (*PTGIS*) Gene Variants

A, Location of 3 identified variants in the primary sequence of *PTGIS*. B, The 3 variants target conserved nucleotides (for c.521 + 1 G>A) or amino acids (for R252Q and A447T) as shown by homological sequence alignment. C, The location of the 2 missense variants on the crystal structure of human *PTGIS*. D, A447T introduces a larger side chain in the ligand-binding space, which is predicted to affect the function of PTGIS. In addition, an alteration from nonpolar alanine to polar threonine will alter the electrostatics of the binding site, which will change enzyme activity.

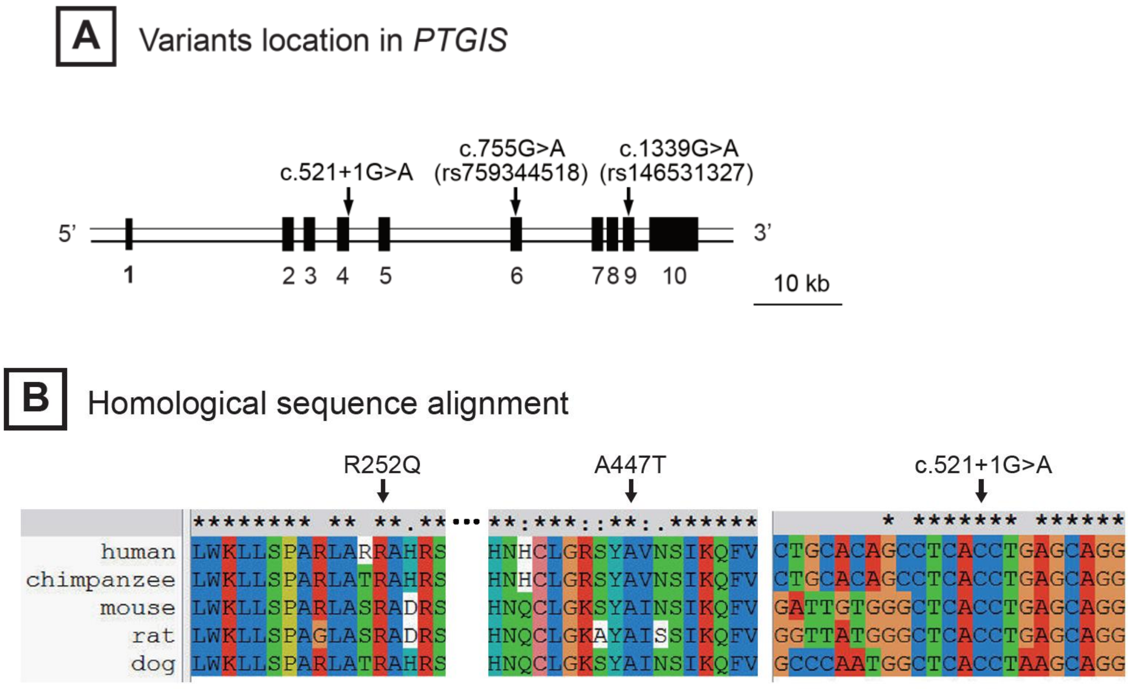

© 2020 Wang X-J et al. *JAMA Cardiology*.

C Crystal structure of human PTGIS

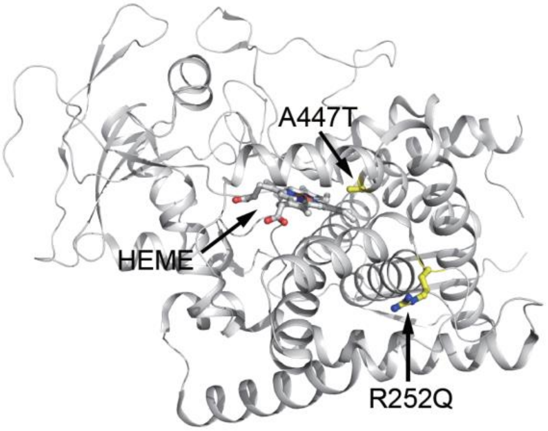

D Predicted functional impact of A447T

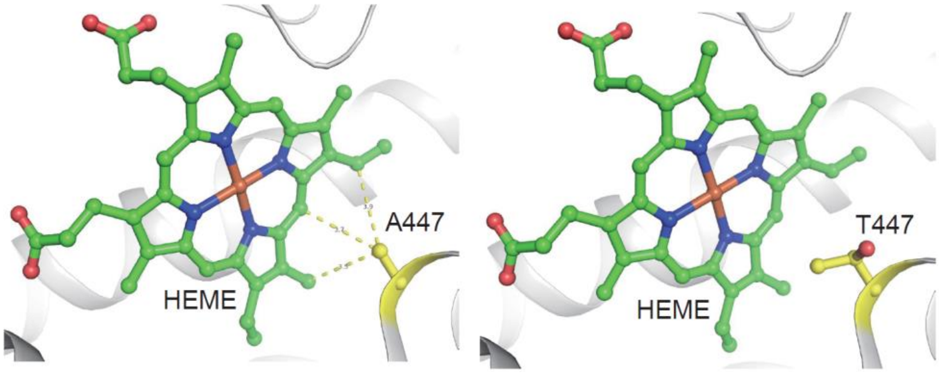

© 2020 Wang X-J et al. *JAMA Cardiology*.

eFigure 3. Verification of the 3 Rare Variants of *PTGIS* in IPAH Cases by Sanger Sequencing

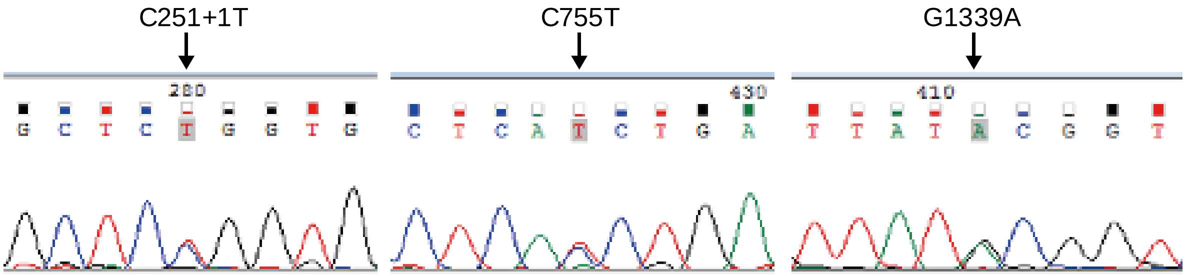

© 2020 Wang X-J et al. *JAMA Cardiology*.

eFigure 4. Analysis of Structure Variants of *PTGIS* in the Replication Cohort by Real-Time PCR

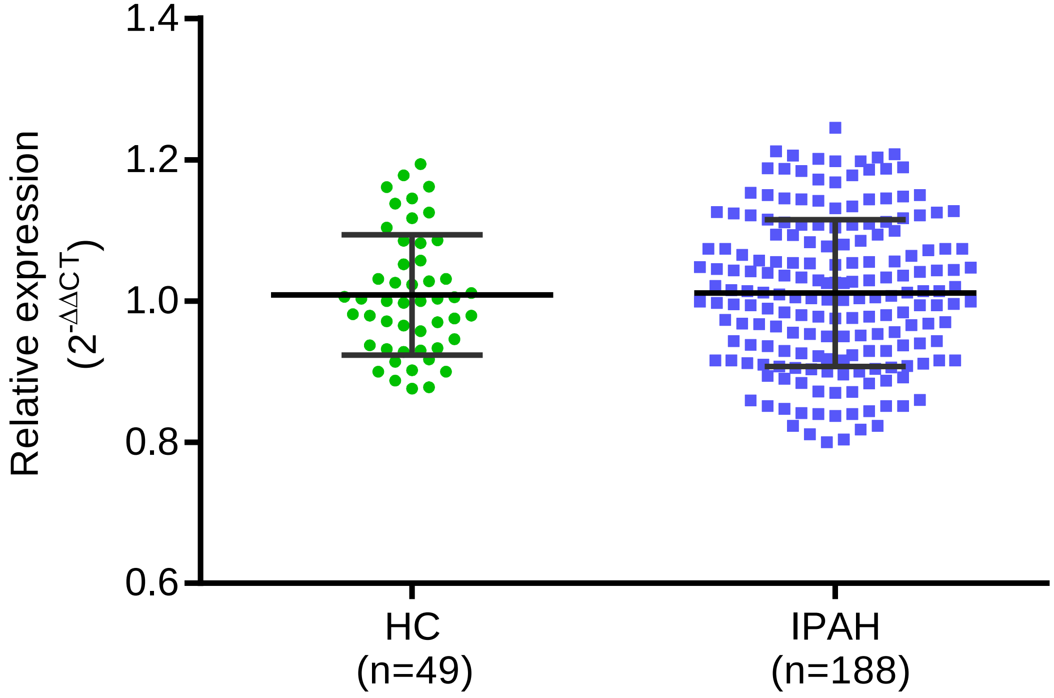

C2 was used for internal reference control. Data were presented as relative fold-change of *PTGIS* to *C2*. HC: healthy control, IPAH: idiopathic pulmonary arterial hypertension.

© 2020 Wang X-J et al. *JAMA Cardiology*.

eFigure 5. Assessment of Genetic Structure of Cases and Controls

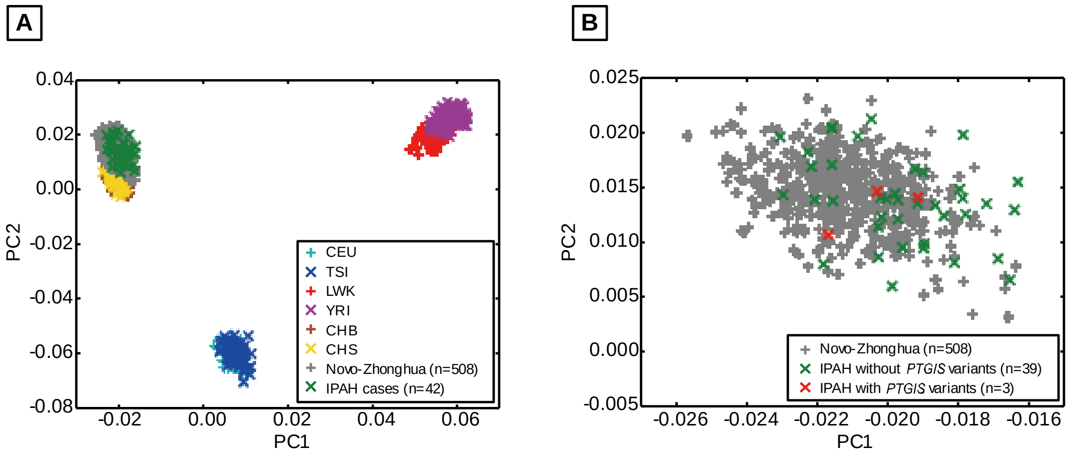

Principal component analysis and comparison of exome-sequencing data of 42 cases, 508 controls, and 1000 genome samples. Panel A demonstrates the presence of 3 groups, corresponding to East Asian descent (yellow), Caucasian descent (blue), and African descent (red). Both the cases and controls were clustered together with East Asian descent. CHB, CHS, CEU, TSI, LWK, YRI denote Han Chinese in Beijing, China (N=103), Southern Han Chinese (N=105), Utah Residents with Northern and Western European Ancestry (N=99), Toscani in Italia (N=107), Luhya in Webuye, Kenya (N=99), and Yoruba in Ibadan, Nigeria (N=108), respectively. Novo-Zhonghua denotes 508 normal controls from Novo-Zhonghua Genomes Database and IPAH Cases denotes 42 patients with IPAH. Panel B demonstrates homogenous population structure for cases and controls.

© 2020 Wang X-J et al. *JAMA Cardiology*.

eFigure 6. Downregulation of *Ptgis* mRNA in the Lungs of MCT and Hypoxia Rat Models

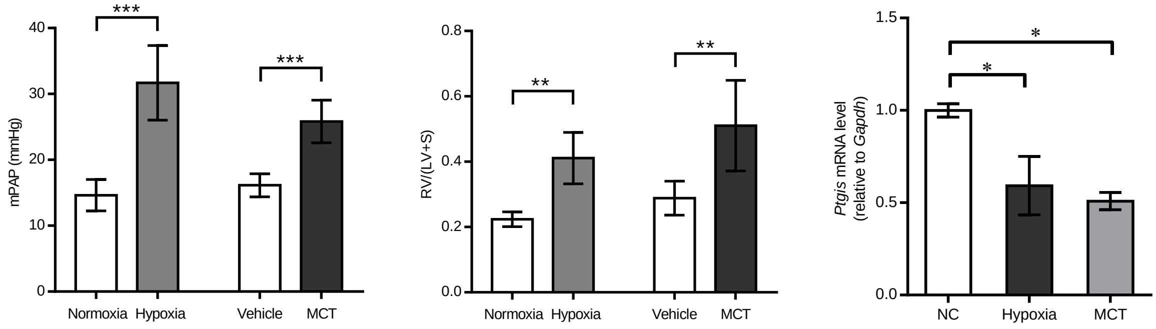

Panel A: Assessment of mean pulmonary artery pressure (mPAP) in hypoxia and monocrotaline (MCT) rat models (n=4–10). Compared with the control group, mPAP increased by 116% in the hypoxia model and 60% in the MCT model (***: $P < .001$; independent t-test). Panel B: Assessment of the right ventricular hypertrophy index (Fulton index) in the hypoxia and MCT rat models (n=4–10). Compared with the control group, RV/(LV+S) increased by 84% in the hypoxia model and 77% in the MCT model (**: $P < .01$; independent t-test). Pancel C: Quantification of the *PTGIS* mRNA level in the lung in the hypoxia and MCT rat models using RT-PCR (n=3–4). The expression of *Ptgis* mRNA decreased by 41% in the hypoxia model and 50% in the MCT model (*: $P < 0.05$; independent *t*-test). All data are presented as means ± SEM.

© 2020 Wang X-J et al. *JAMA Cardiology*.

eFigure 7. Downregulation of *Ptgis* mRNA in the Lungs of Sugen Plus Hypoxia Rat Models

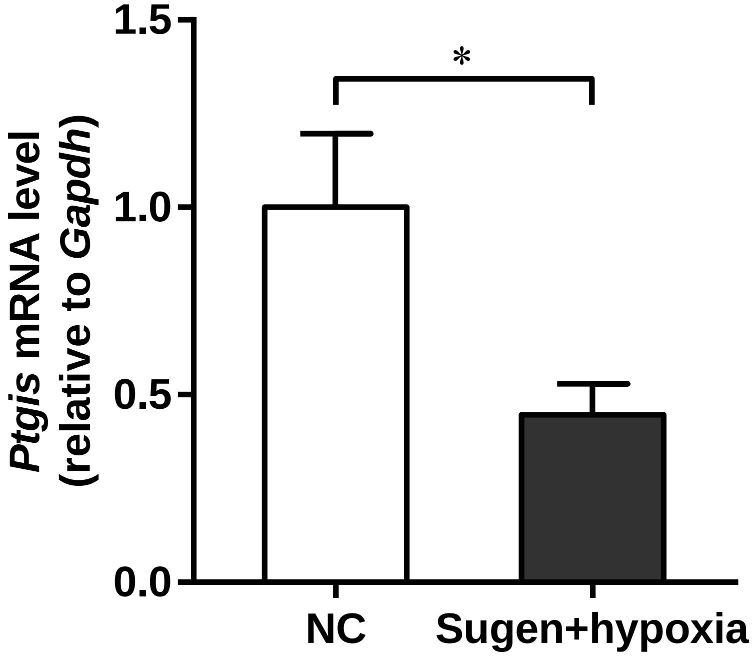

Quantification of the *Ptgis* mRNA level in the lungs of sugen+hypoxia rat models using RT-PCR. *Gpadh* was used as an internal control. Compared with the control group (n=7), the expression of *Ptgis* decreased by 55% in the lung of Sugen/hypoxia rat models (n=7) (*: $P < 0.05$; independent *t*-test). Data are presented as means ± SEM.

© 2020 Wang X-J et al. *JAMA Cardiology*.

eFigure 8. Blueprint of the Minigene Plasmid Construction

Construct Design (In Fusion)

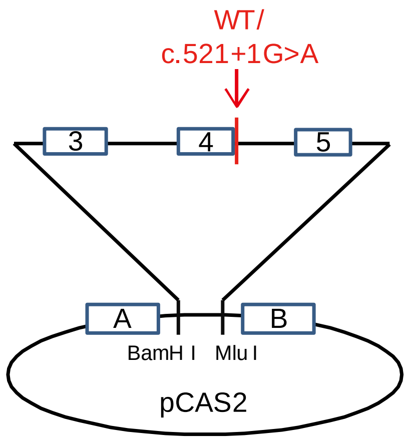

DNA fragments containing exons 3~5 and the flanking intronic sequences of *PTGIS* was amplified from the genome DNA of the patient heterozygous for the variant c.521+1G>A. The DNA fragments were inserted to the linearized (BamHI-MluI digested) minigene vector pCAS2 by homologous recombination. The arrow indicated the site for c.521+1G>A.

© 2020 Wang X-J et al. *JAMA Cardiology*.

eFigure 9. Protein Blot Analysis

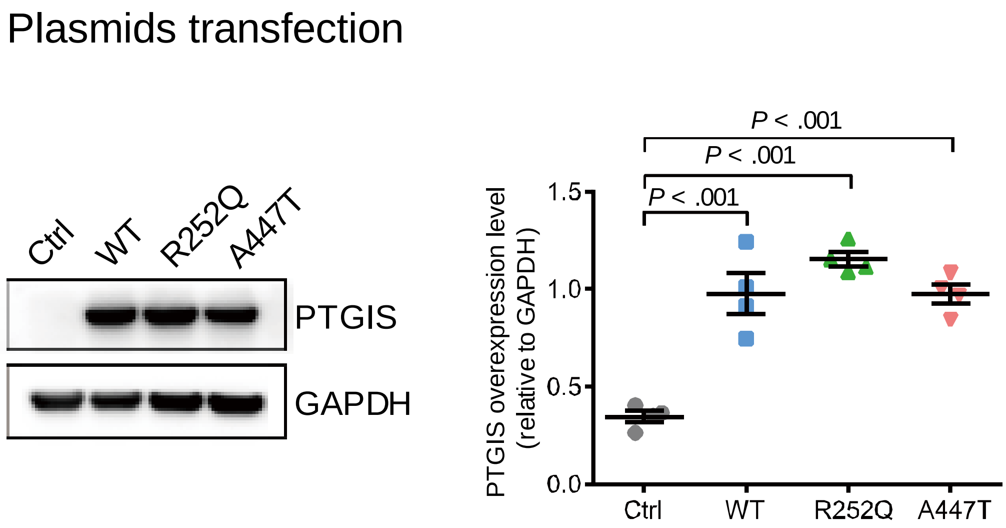

Immunoblotting of pulmonary microvascular endothelial cells (PMECs) transfected with plasmids for the wild-type *PTGIS* or the two missense variants (R252Q and A447T). PMECs transfected with empty vector were used as a negative control, and GAPDH served as a loading control.

© 2020 Wang X-J et al. *JAMA Cardiology*.

eFigure 10. Cell Viability Assay

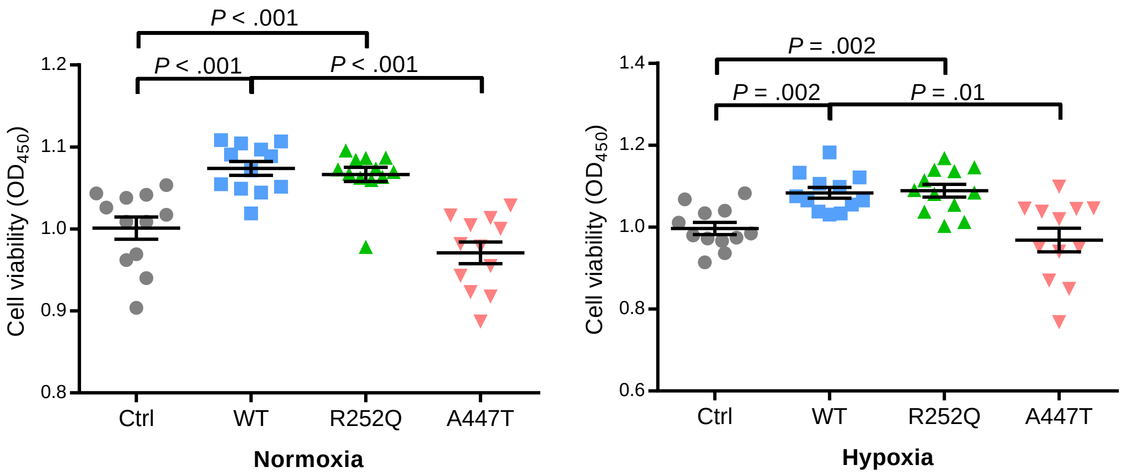

The cell viability was analyzed in pulmonary microvascular endothelial cells (PMECs) transfected with plasmids for the wild-type *PTGIS* or the two missense variants (R252Q and A447T) under normoxia (left panel) and hypoxia conditions (right panel). PMECs transfected with empty vector were used as a negative control,

© 2020 Wang X-J et al. *JAMA Cardiology*.

eFigure 11. Representative Images of Tube Formation From Pulmonary Microvascular Endothelial Cells (PMECs) Transfected With the Plasmids for Wild-type (WT) and 2 Variants

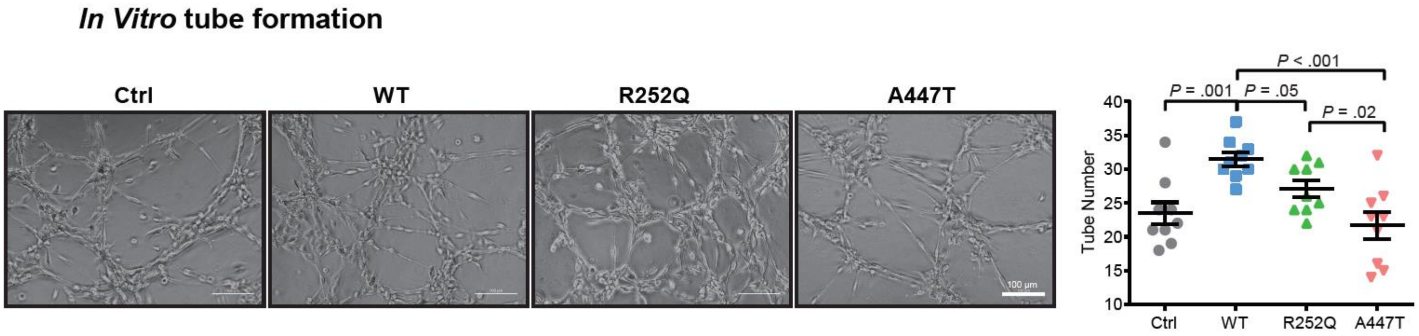

© 2020 Wang X-J et al. *JAMA Cardiology*.

eFigure 12. Conceptual Representation of the Role of *PTGIS* Rare Variants in PAH

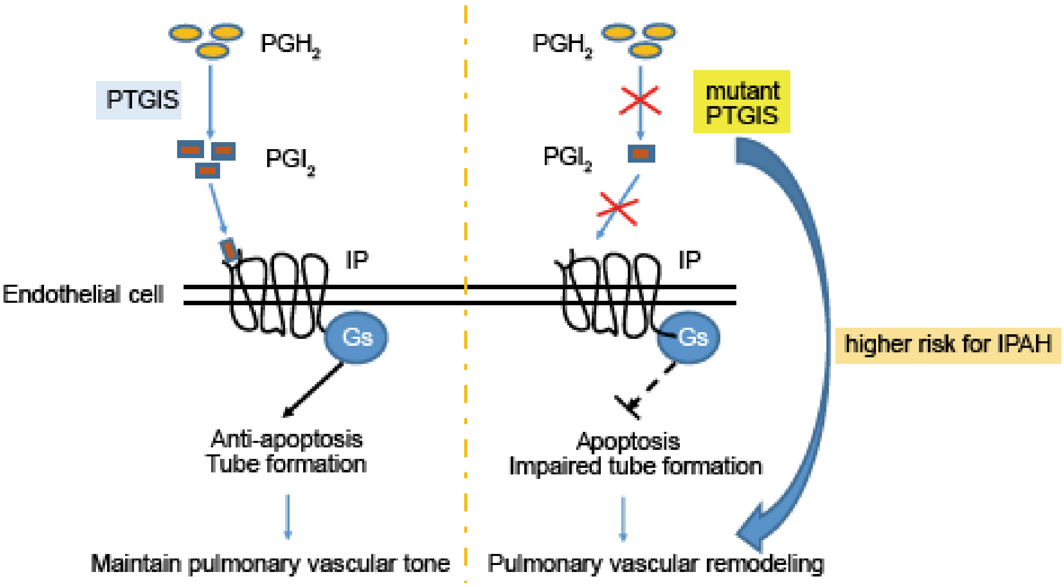

At the physiological conditions, PTGIS catalyzes PGH_2 into PGI_2, maintaining the pulmonary vascular tone (left panel). The rare variants of *PTGIS* result in reduction on biosynthesis of PGI_2 and deleterious effects on the function of pulmonary endothelium cells, contributing to higher risk for development of idiopathic pulmonary arterial hypertension (right panel).

© 2020 Wang X-J et al. *JAMA Cardiology*.

eMethods. Sequencing and Analysis

Whole-genome sequencing and variant prioritization

WGS was performed at WuXi Apptech (Shanghai, China) using a PCR-free library preparation and paired-end (2 × 150 bp) sequencing on the HiSeq × Ten platform (Illumina, Inc., San Diego, CA, USA) to yield a minimum of 35 × coverage. Prior to sequencing, samples were randomized to minimize batch effects. Genomes were aligned to the human genome reference sequence (hg19 build) with Burrows-Wheeler alignment. The HaplotypeCaller was used to call SNVs, insertions, and deletions (indels). Both the aligned and unaligned reads were delivered in binary sequence alignment/map format together with variant call format (VCF) files. VCF files were generated per individual, and variants failed the quality filter were excluded.

We conducted 4 steps to process the VCF files and prioritize variants that were likely to be deleterious. Initially, these VCF files were annotated with the ANNOVAR software to obtain comprehensive annotations for each variant. The used annotation packages from ANNOVAR included refGene, popfreq_all_20150413, dbnsfp30a, snp138 and KG_Chinese. KG_Chinese is a customized annotation that contains variant frequency with respect to 208 Chinese individuals from 1000 Genomes Project (phase 3). Next, we excluded variants in intronic (other than variants considered to be splicing variants and located 2 bp to the junction) and intergenic regions. Thus, only variants in exonic regions and splicing sites were included for further analysis. In the third step, we employed variant frequency filters to extract rare variants. The two filters used were variant frequency in all populations and in Chinese population. For variant frequency in all populations, these were excluded if corresponding minor allele frequencies were larger than 0.5% in any one population from the public databases, including db SNP138, 1000 Genomes Project (phase 3), Exome Sequencing Project, and genome Aggregation Database (gnomAD). For variant frequency in Chinese population, these were excluded if presented in Chinese population from the KG_Chinese annotation. Lastly, functional prediction scores were employed to prioritize deleterious variants. For variants in exonic regions, 11 open-access software tools including SIFT, PolyPhen2 HDIV, PolyPhen2 HVAR, LRT, MutationTaster, MutationAccessor, FATHMM, PROVEAN, FATHMM-MKL, MetaSVM, and MetaLR were used. Variants were classified as probably damaging if the variants were predicted by at least six software tools to be deleterious. The default thresholds were used for all the 11 software tools. For variants in splicing sites, two splice-site prediction programs, including dbscSNV[1] and SPIDEX[2], were used to predict the effect of variants on mRNA splicing.

For the *PTGIS* gene copy number variants (CNVs) and structure variants (SVs) analysis, the Control-FREEC[3] was used to analyze the copy number variants (CNVs) of *PTGIS* in the discovery cohort. Both CREST[4] and BreakDancer[5] were used on each WGS sample to assess the structure variants (SVs) of *PTGIS*. The CNVs and SVs covering the coding regions of *PTGIS* were confirmed by Integrative Genomics Viewer[6], which was also used to check whether there were missing indels. The homogenous population structure of cases and controls was defined genetically by means of principle-component analysis of common variants that were sequenced.

Sanger sequencing validation

Complete sequencing of the 10 exons and splicing sites of the *PTGIS* gene was performed in the replication sample on an ABI 3730 automated sequencer (Applied Biosystems, Courtaboeuf, France) using specific primers (Supplementary Table S1).

Pulmonary hemodynamics measurement and vasoreactivity testing

A quadric-lumen 7F (for adults) or 5F (for children) Swan-Ganz catheter (Edwards Lifesciences World Trade Co. Ltd, Irvine, CA, USA) was advanced into the pulmonary artery to measure pulmonary hemodynamics at baseline,

© 2020 Wang X-J et al. *JAMA Cardiology*.

including the mean pulmonary artery pressure (mPAP), pulmonary arterial wedge pressure (PAWP), right atrial pressure (RAP), pulmonary vascular resistance (PVR), and mixed venous oxygen saturation (Svo2). Cardiac output (CO) was measured by the standard thermodilution technique. Cardiac index (CI) was calculated as cardiac output divided by body surface area.

After evaluation of baseline parameters, acute vasoreactivity testing was performed according to previously described protocols.[7]. Iloprost (5μg, Ventavis®; Bayer–Schering Pharma, Berlin, Germany) was delivered by a PARI LC STAR nebulizer (PARI GmbH, Starnberg, Germany) driven by a PARI TurboBOY-N compressor (PARI GmbH) for 15min. Pulmonary hemodynamics was recorded after the administration of illoprost. The change in PVR was calculated as: 100 x ($PVR_{\text{after iloprost inhalation}}$ – PVR_{baseline}) / PVR_{baseline}. The change in CI was calculated as: 100 x ($CI_{\text{after iloprost inhalation}}$ – CI_{baseline}) / CI_{baseline}.

Analysis of structure variants of *PTGIS* by quantitative real-time PCR (qRT-PCR)

Twelve SVs have been reported in the *PTGIS* gene interval according to the DGV (Databases of Genome Variants) site, all of which were deletions. SYBR green-based quantitative PCR (Roche Life Science, Indiana. USA) was used to detect SVs of *PTGIS* in the replication cohort on viiA7 Real-Time PCR System (Applied Biosystems, CA, USA). Melting curve analysis was performed to confirm the specificity of the PCR products. *C2* gene was used as internal control. The primers for *PTGIS* gene are Forward: CCTTCCTTGCCTTATCAAATGGTG; Reverse: CCAAGGCATACCCCAACCAG. The primers for *C2* gene are Forward: CAATTCAGGTCAGGTGATAACTCAGTA; Reverse: GCCAGGTTTAGAATGTTTGTCTAAGTC. All experiments were repeated twice in triplicate.

Animal studies

All procedures were performed according to the Guide for Care and Use of Laboratory Animals published by the US National Institutes of Health (NIH Publication 85-23, revised 1996), and approved by the Institutional Committee for Use and Care of Laboratory Animals of FuWai Hospital (Beijing, China).

Monocrotaline (MCT)-induced pulmonary hypertension

Male SD rats (180-220g, body weight) from the Beijing Vital River Laboratory Animal Technology Co., Ltd. (Beijing, China) were randomized into treatment group and control group. They were administered a single subcutaneous injection of 50 mg/kg MCT (Sigma-Aldrich, St. Louis, MO, USA) or sterile saline (vehicle control). Hemodynamic and right ventricular hypertrophy were assessed 3 weeks after MCT injection. Rats were euthanized and tissues were collected for analysis.

Chronic hypoxia-induced pulmonary hypertension

Male SD rats (180-220g, body weight) were placed in a hypoxic chamber containing 10% O2. After continually exposure to hypoxia for 3 weeks, hemodynamics and right ventricular hypertrophy were assessed. Then the rats were euthanized. The heart and lung were harvested for further research.

Sugen/hypoxia-induced pulmonary hypertension

The lung cDNA of Sugen/hypoxia-induced pulmonary hypertension rat models were generously offered by Dr. John Y.-J. Shyy from Department of Medicine, University of California, San Diego. This rat model was constructed in Dr, Shyy's lab by the following method. Male rats (200-250g, body weight) received one dose of 20mg/kg SU5416 (Sigma-Aldrich, St. Louis, MO, USA) by subcutaneously injection and were housed under hypoxic conditions (10% O_2) for 3 weeks. After hypoxia exposure, the rats were put back to the room air for another 2 weeks. Rats receiving DMSO for the same duration were negative controls. At the end of the experiments, the animals were used for

© 2020 Wang X-J et al. *JAMA Cardiology*.

hemodynamic measurements and the tissues were harvested.

Real-time PCR

Total RNA was extracted using TRIzol Reagent (Invitrogen, MA, USA) according to the manufacturer's instructions. cDNA was synthesized using SuperScript III Reverse Transcriptase (Invitrogen). Expression of *Ptgis* mRNA was assessed using the SYBR green-based quantitative real-time PCR. Levels of mRNA were normalized to *Gapdh* for quantification using the $2^{-\Delta\Delta CT}$ comparative quantification method.

Plasmid construction

For the minigene constructs, a DNA fragment of 8762 bp encompassing exons 3~5 and the flanking intronic sequences of *PTGIS* was amplified from the genome DNA of the patient heterozygous for this variant, using the primer pair *PTGIS*-InFu-Ex3-F (5'-AAGAAGTGCAGGATCCAGTCCTAGTCCTGCCACTTCTC-3') and *PTGIS*-InFu-Ex5-R (5'-TCAAAACAAGACGCGTTCTGGGTCTTTCCATCCTCC-3'). On the 5' ends of both primers were 15 bp extensions complementary to each end of the linearized expression minigene vector pCAS2, which was generated by digestion of the restriction enzymes *BamH I* and *Mlu I*. Gel-purified PCR product was recombined into the linearized pCAS2, using an In-Fusion HD Cloning Kit (TAKARA; Kusatsu, Shiga, Japan) according to the user manual (Fig. 2A). The resultant wild-type and mutant minigenes were confirmed by Sanger sequencing, and referred to as pCAS2-*PTGIS*-Ex3-5-WT and pCAS2-*PTGIS*-Ex3-5-VAR, respectively.

For the two missense mutations plasmid construction, human wild-type (WT) *PTGIS* cDNA (1500 bp, NM_000961.3, Tianyi Huiyuan, China) was subcloned into the *SalI/KpnI* site of pCMV-HA plasmid. The two mutant plasmids (R252Q and A447T) were constructed based on the WT plasmid using the QuickChange Mutagenesis Kit (Stratagene, La Jolla, CA, USA). All constructs were sequenced to confirm the integrity and the presence of mutations. All plasmids were transfected into cells using Lipofectamine 3000 reagent (Invitrogen).

Cell culture

HEK293T cells were grown in a 5% CO_2 incubator at 37°C in Dulbecco's modified Eagle's medium (DMEM, from Union Cell Resource Center, China) supplemented with 10% fetal bovine serum (Gibco, NY, USA). Cells were seeded at 80% confluence in a 6-well plate 12 h before transfection. After transfection with WT and mutant minigene plasmids for 48 h, cells were lysed for analysis of mRNA transcripts.

Human pulmonary microvascular endothelial cells (PMECs, Sciencell, CA, USA) were maintained in endothelial cell (EC) growth medium (Sciencell) and used between passages 4 and 6. Transfected PMECs were treated with 10 μM arachidonic acid (Sigma, St. Louis, USA) in 1% EC medium (1% fetal bovine serum with penicillin/streptomycin) for 24 h for the in vitro tube-formation assay, the cell viability assay, and measurement of 6-keto-$PGF_{1\alpha}$ concentrations. For hypoxia treatment, transfected PMECs were maintained at 1% O_2 for 24 h.

Analysis of mRNA transcripts from transfected HEK293T cells

HEK293T cells were washed twice with PBS 48 h after transfection. Total RNA was extracted using a standard procedure with TRIzol and chloroform. RT-PCR was performed immediately after RNA extraction with PCR primer pair *PTGIS*-minigene-RT-F (5'-GTGATGAAAAGGCCAGGATG-3') and *PTGIS*-minigene-RT-R (5'-TTGAGGAGGCTCTATCCCAC-3') located in *PTGIS* exons 3 and 5, respectively. Splice products of each well were visualized by gel electrophoresis. DNA fragments of interest were gel-purified and verified by Sanger sequencing.

Measurement of 6-Keto-$PGF_{1\alpha}$

Due to the short half-life of prostacyclin (42 seconds),[8] the amount of prostacyclin produced by PMECs was monitored by measurement of its stable metabolite, 6-keto-$PGF_{1\alpha}$, in the culture supernatant using ELISA Kit (Cayman,

© 2020 Wang X-J et al. *JAMA Cardiology*.

Michigan, USA).

Tube formation assay

Endothelial tube formation was assessed using the Matrigel assay (BD Biosciences, NJ, USA). Transfected HPMECs treated with arachidonic acid were seeded at a density of 5×10^3/well on 96-well plates precoated with 50 µl growth-factor-reduced Matrigel followed by incubation at 37°C for 6 h. Tube formation was then examined under a phase-contrast microscope (Leica, Wetzlar, Germany).

Cell viability

Transfected PMECs were treated with 10 µM arachidonic acid for 24 h and maintained under normoxic or hypoxic conditions. Cell viability was then assessed using Cell Counting Kit-8 (Dojindo Laboratories, Shanghai, China).

Caspase 3/7 activity assay

Transfected PMECs were stimulated with 10 ng/ml tumor necrosis factor-□ and 20 µg/ml cycloheximide for 3 h to induce apoptosis. The activity of caspase 3/7 was determined using the Caspase-Glo 3/7 Assay according to the manufacturer's instructions.

Western blotting

PMECs were lysed by RIPA buffer (containing phosphatase and protease inhibitors; Roche, Basel, Switzerland), centrifuged, and boiled with 5 × sodium dodecyl sulfate polyacrylamide (SDS) loading buffer at 100°C for 10 min. Cell lysates were separated on 12% (wt/vol) SDS polyacrylamide electrophoresis gel, and proteins were transferred onto polyvinylidene difluoride membranes. The membranes were blocked with 5% skim milk for 1 h at room temperature, and then incubated with primary antibodies in 5% bovine serum albumin (BSA) overnight at 4°C then subsequently probed with secondary antibodies in 5% BSA for 1 h. The membranes were imaged using the chemiluminescence reagent FluorChem M (ProteinSimple, CA, USA).

© 2020 Wang X-J et al. *JAMA Cardiology*.

eTable 1. Filtering Strategy to Identify Rare Genetic Variants in Whole-Genome Sequencing Data

Filtering options	Number of variants
Total variants of high quality	12,101,922
Variants at exon or splicing sites[a]	68,634
Variants at nsSNV/splice site[b]	33,952
Rare variants[c]	6938
Variants potentially hazardous to gene functions[d]	1986

[a]: Variants located in exons or within 2 bp of a splicing site

[b]: Variants belonging to non-synonymous, splice acceptor or donor site, or frameshift coding indels.

[c]: The variants were filtered if corresponding minor allele frequencies were more than 0.5% in any of the following genetic databases: db SNP138, 1000 Genome Project, Exome Sequencing Project (ESP), and the genome Aggregation Database (gnomAD) . The variants were also excluded if presented in Chinese population from the 1000 Genomes Project.

[d]: The potential pathogenic effects were analyzed by 11 *in silico* tools. The variants were considered deleterious by at least 6 of the 11 predictors.

© 2020 Wang X-J et al. *JAMA Cardiology*.

eTable 2. Candidate Genes Identified Via WGS in the Discovery Cohort							
**Gene ID**	**Chromosome location**	**Gene name**	**Gene ontology**	**Associated diseases**	**Number of individuals**	**Number of variants**	**Expression level in the lung[a]**
5740	20q13.13	*PTGIS*	Mono-oxygenase activity, iron ion binding, protein binding, prostaglandin-I synthase activity, oxidoreductase activity	Pulmonary hypertension, hypertension, cerebral infarction	3	2	141
9344	16p11.2	*TAOK2*	Protein serine/threonine kinase activity, ATP binding, kinase activity	No	4	4	11
26058	2q37.1	*GIGYF2*	Protein binding, poly(A) RNA binding, proline-rich region binding, cadherin binding involved in cell–cell adhesion.	Parkinson's disease	4	3	14
23499	1p34.3	*MACF1*	Actin binding, calcium ion binding, protein binding, microtubule binding, cytoskeletal protein binding.	No	4	4	373
2975	16p12.1	*GTF3C1*	Transcription factor activity, core RNA polymerase III binding.	No	3	2	119
83872	1q25.3-q31.1	*HMCN1*	Calcium ion binding	Macular degeneration	3	3	25
23195	6q15	*MDN1*	Nucleus, nucleolus, cytoplasm, membrane, preribosome, large subunit precursor	No	3	3	5
273	7p14.1	*AMPH*	ARF guanyl-nucleotide exchange factor activity, protein binding, phospholipid binding	No	3	3	4
6350	22q13.33	*SBF1*	Protein tyrosine/serine/threonine phosphatase activity, rab guanyl-nucleotide exchange factor activity.	Charcot-Marie-Tooth disease, type 4B3	3	2	12

© 2020 Wang X-J et al. *JAMA Cardiology*.

Gene ID	Chromosome location	Gene name	Gene ontology	Associated diseases	Number of individuals	Number of variants	Expression level in the lung[a]
55567	16p12.3	*DNAH3*	Motor activity, microtubule motor activity, ATP binding.	No	3	3	7
2318	7q32.1	*FLNC*	Protein binding, cytoskeletal protein binding, ankyrin binding.	Cardiomyopathy, familial hypertrophic; cardiomyopathy, familial restrictive	3	4	12
21	16p13.3	*ABCA3*	Transporter activity, ATP binding, ATPase activity.	Surfactant metabolism dysfunction, pulmonary	3	3	856
463	16q22.2-q22.3	*ZFHX3*	Core promoter sequence-specific DNA binding, DNA binding, transcription factor activity	Prostate cancer	3	3	5
7253	14q31.1	*TSHR*	G-protein coupled receptor activity, thyroid-stimulating hormone receptor activity, protein binding	Hyperthyroidism, familial gestational; thyroid adenoma, hyperfunctioning, somatic	3	3	5
4363	16p13.11	*ABCC1*	Transporter activity, ATP binding, ATPase activity	No	3	3	9

Abbreviations: ARF denotes auxin response factor; ATP, adenosine triphosphate; IPAH, idiopathic pulmonary arterial hypertension; and WGS, whole-genome sequencing.

[a]: the data of candidate genes expression in the human lung tissue were taken from the Biogps database (http://biogps.org/).

© 2020 Wang X-J et al. *JAMA Cardiology*.

eTable 3. The PCR Primers for Amplifying the 10 Exons of the *PTGIS* Gene

Exon	Primer	Sequence (5' to 3')	Base number	Product size (bp)
Exon 1	F	CTGGGGGGAGCAGGGTTTCT	20	558
	R	GCCAGACTCGGACTTGTCGC	20	
Exon 2	F	GCTCCATTCAGATTACCTCC	20	389
	R	TGGATAGAAAGTTGGCTCGAC	21	
Exon 3	F	GAGTGATGGTCCCTACCTCA	20	459
	R	TGTCTTTACCGAACATTTGCT	21	
Exon 4	F	GATGGGAACTATTGTTGACGA	21	466
	R	CTACAGTCTTTGCTTTGGGT	20	
Exon 5	F	TGCTCACTGGGTGTATGAATG	21	357
	R	ATTGGATGTCTTTCTGCCTGT	21	
Exon 6	F	AGTGACATTCAGAAGACCCT	20	505
	R	AAAGGAAGTCAGGGAACTGG	20	
Exon 7	F	GGGTGTTTGGCAGCAGTGG	19	349
	R	GGGGTCCATGATGACGCAGT	20	
Exon 8	F	CCACCTTCCCTCACAACTT	19	497
	R	ATCTGGCTCATCCCAAAGTC	20	
Exon 9	F	ACAGGCATACACACAACACA	20	564
	R	CCTCTGCCAAACCATTCTCC	20	
Exon 10	F	CTGGCAAATGTGGTGAGCA	19	444
	R	GAAAAGCAGGGAAGTGGTAATG	22	

© 2020 Wang X-J et al. *JAMA Cardiology*.

eTable 4. Rare Variants in the *PTGIS* Gene

Chromosome location	Nucleotide change[a]	Amino acid change[b]	SNP	Exon	Function	MAF (KG_Chinese) [c]	MAF (PopFreq Max) [d]	GERP++RS[e]	Mutation-Taster[f]	Poly-Phen2[f]	SIFT**	SPIDEX_dPST Percentile[g]	dbscSNV_ada Score[f]
chr20:48160841	c.521+1G>A	_	_	4	Splicing	0	0	5.04	1	_	_	0.02	0.99
chr20:48140695	c.755G>A	p.Arg252Gln	Rs759344518	6	Missense	0	0.002	4.29	0.70	0.98	0.53	_	_
chr20:48127584	c.1339G>A	p.Ala447Thr	rs146531327	9	Missense	0	0.003	5.26	1	1	0	_	_

Abbreviation: SNP denotes single nucleotide polymorphism.

[a]: Abbreviations are in accord with nomenclature guidelines as recommended by the Human Genome Variation Society (http://varnomen.hgvs.org). The letter c. is used to indicate coding DNA, where nucleotide 1 is the A of the ATG translation initiation codon. The variants are annotated by human *PTGIS* cDNA (Genebank NM_000961.3).

[b] p. is used to indicate change at the protein level.

[c]: The minor allele frequency in the Chinese cohort of the 1000 Genomes dataset (www.1000genomes.org). This cohort includes two populations from China: CHS (105 individuals from China South) and CHB (103 individuals from Beijing).

[d]: Maximum minor allele frequency among the following public genomic datasets, including dbSNP (v139), 1000 Genome Project (Phase 3), NHLBI Exome Variant Server (ESP), genome Aggregation Database (gnomAD).

[e]: The larger the score, the more conserved the site.

[f]: Scores range from 0 to 1. The larger the score the more likely the SNP has a damaging effect.

[g]: Scores range from 0 to 1. The smaller the score the more likely the SNP has a damaging effect.

© 2020 Wang X-J et al. *JAMA Cardiology*.

eTable 5. Clinical Characteristics of the 14 Patients With *PTGIS* Rare Variants

Patient ID	*PTGIS* variant	Gender	Age at diagnosis, yrs	WHO FC	6MWD, m	mPAP, mmHg	PVR, Wood units	CI, liters/min/m^2
34	c.1339G>A	Female	25	III	327	57	24.9	1.2
237	c.1339G>A	Female	46	III	405	52	12.3	2.2
811	c.1339G>A	Female	28	III	444	53	10.8	2.6
1306	c.1339G>A	Female	26	II	445	45	8.2	3.2
1726	c.1339G>A	Male	70	II	324	52	9.3	3.4
B16	c.1339G>A	Female	26	I	555	35	7.6	2.7
B377	c.1339G>A	Female	23	III	437	63	15.3	2.4
B2140	c.1339G>A	Female	32	III	344	40	7.1	4.7
1819	c.755G>A	Female	66	III	230	69	11.6	3.1
2378	c.755G>A	Female	17	II	470	31	4.2	2.7
2379	c.755G>A	Female	17	I	580	50	4.0	4.7
B2402	c.755G>A	Male	44	III	323	44	17.1	1.5
B1380	c.755G>A	Female	26	III	335	77	21.1	1.9
1654	c.521+1G>A	Female	22	IV	NS	70	18.9	2.5

Abbreviations: 6MWD denotes 6-minute walking distance; mPAP, mean pulmonary artery pressure; PVR, pulmonary vascular resistance, CI, Cardiac Index, and WHO FC, World Health Organization functional class.

© 2020 Wang X-J et al. *JAMA Cardiology*.

eTable 6. The Influence of *PTGIS* Rare Variants on Pulmonary Vascular Response to Iloprost

**Clinical Characteristics**	**IPAH without *PTGIS* rare variants (n=194)**	**IPAH with *PTGIS* rare variants (n=12)**	***P* value**
Age at diagnosis, mean (SD),yrs	34 (15)	33 (18)	0.70
Female, No. (%)	145 (76)	10 (83)	0.73
PVR at baseline, mean (SD), Wood units	15.02 (6.80)	13.63 (6.94)	0.42
PVR after iloprost inhalation, mean (SD), Wood units	12.82 (6.57)	10.10 (7.17)	0.15
Changes in PVR, mean (95% CI), %	-15.28 (-18.33, -12.23)	-32.87 (-48.14, -17.59)	**0.01**
CI at baseline, mean (SD), L/min/m^2	2.61 (1.04)	2.64 (0.93)	0.52
CI after iloprost inhalation , mean (SD), L/min/m^2	2.81 (1.19)	3.13 (1.03)	0.12
Changes in CI, mean (95% CI), %	8.89 (6.15, 11.64)	20.21 (7.58, 32.85)	**0.04**

IPAH denotes idiopathic pulmonary arterial hypertension; CI, cardiac index; PVR, pulmonary vascular resistance; Changes in PVR = 100 *($PVR_{after\ iloprost\ inhalation}$ – $PVR_{baseline}$) / $PVR_{baseline}$; Changes in CI = 100 *($CI_{after\ iloprost\ inhalation}$ – $CI_{baseline}$) / $CI_{baseline}$. 95% CI: 95% confidence interval.

© 2020 Wang X-J et al. *JAMA Cardiology*.

eReferences.

1. Jian X, Boerwinkle E, Liu X. In silico prediction of splice-altering single nucleotide variants in the human genome. *Nucleic Acids Res*. 2014;42(22):1353413544.
2. Xiong HY, Alipanahi B, Lee LJ, et al. RNA splicing. The human splicing code reveals new insights into the genetic determinants of disease. *Science*. 2015;347(6218):1254806.
3. Boeva V, Popova T, Bleakley K, et al. Control-FREEC: a tool for assessing copy number and allelic content using next-generation sequencing data. *Bioinformatics*. 2012;28(3):423-425.
4. Wang J, Mullighan CG, Easton J, et al. CREST maps somatic structural variation in cancer genomes with base-pair resolution. *Nat Methods*. 2011;8(8):652-654.
5. Chen K, Wallis JW, McLellan MD, et al. BreakDancer: an algorithm for high-resolution mapping of genomic structural variation. *Nat Methods*. 2009;6(9):677-681.
6. Robinson JT, Thorvaldsdóttir H, Winckler W, et al. Integrative genomics viewer. *Nat Biotechnol*. 2011;29(1):24-26.
7. Jing ZC, Jiang X, Han ZY, et al. Iloprost for pulmonary vasodilator testing in idiopathic pulmonary arterial hypertension. *Eur Respir J*. 2009;33(6):1354-1360.
8. Cawello W, Schweer H, Müller R, Bonn R, Seyberth HW. Metabolism and pharmacokinetics of prostaglandin E1 administered by intravenous infusion in human subjects. Eur J Clin Pharmacol 1994;46(3):275-277.

© 2020 Wang X-J et al. *JAMA Cardiology*.
